# Altered Spontaneous Brain Activity and Network Property in Patients With Congenital Monocular Blindness

**DOI:** 10.3389/fneur.2022.789655

**Published:** 2022-02-24

**Authors:** Jingwen Ding, Xiaoxia Qu, Jing Cui, Jie Dong, Jian Guo, Junfang Xian, Dongmei Li

**Affiliations:** ^1^Beijing Ophthalmology & Visual Science Key Lab, Department of Ophthalmology, Beijing Tongren Hospital, Capital Medical University, Beijing, China; ^2^Department of Radiology, Beijing Tongren Hospital, Capital Medical University, Beijing, China

**Keywords:** monocular blindness, congenital microphthalmia, resting-state functional MRI, amplitude of low frequency fluctuation, brain network

## Abstract

Individuals with congenital monocular blindness may have specific brain changes since the brain is prenatally deprived of half the normal visual input. To explore characteristic brain functional changes of congenital monocular blindness, we analyzed resting-state functional MRI (rs-fMRI) data of 16 patients with unilateral congenital microphthalmia and 16 healthy subjects with normal vision to compare intergroup differences of amplitude of low frequency fluctuations (ALFFs), functional connectivity (FC), and network topolgoical properties. Compared with controls, patients with microphthalmia exhibited significantly lower ALFF values in the left inferior occipital and temporal gyri, superior temporal gyrus, inferior parietal lobe and post-central gyrus, whereas higher ALFF in the right middle and inferior temporal gyri, middle and superior frontal gyri, left superior frontal, and temporal gyri, such as angular gyrus. Meanwhile, FC between left medial superior frontal gyrus and angular gyrus, FC between left superior temporal gyrus and inferior parietal lobe and post-central gyrus decreased in the patients with congenital microphthalmia. In addition, a graph theory-analysis revealed increased regional network metrics (degree centrality and nodal efficiency) in the middle and inferior temporal gyri and middle and superior frontal gyri, while decreased values in the inferior occipital and temporal gyri, inferior parietal lobule, post-central gyrus, and angular gyrus. Taken together, patients with congenital microphthalmia had widespread abnormal activities within neural networks involving the vision and language and language-related regions played dominant roles in their brain networks. These findings may provide clues for functional reorganization of vision and language networks induced by the congenital monocular blindness.

## Introduction

Although visual function integrity is considered essential to the development and efficacy of cognitive function (1), congenitally blind individuals overall present perceptual, cognitive, and social skills comparable with those of sighted ones (2). Findings from neruoimaging studies have contributed to understand not only the cross-modal plastic changes that occur in the “visual” regions when deprived of vision, but also the role of visual experience on the development of the brain morphological and functional architecture (2). Abundant evidence supports that an early and prolonged absence of vision induces an adaptive reshaping of the brain that spreads beyond the visual areas (3–5). The plastic rearrangements occur outside the visually deprived occipital cortex, such as cortical, subcortical, and white matter (WM) structures (6).

Monocular blindness occurring early in life could be due to genetic anomalies, tumor or injury to the developing visual system, which results in premature loss of stereoscopic binocular vision and a decrease in peripheral visual fields. Challenges of monocularity are imposed on patients both physically and emotionally (7, 8). The impact of unilateral blindness on the functional status and wellbeing is more subtle and less well-understood when compared with bilateral blindness (9). Research on the structural and physiological consequences following early monocular enucleation found that losing binocularity early leads to a dissociation in form perception and motion processing. Low- to mid-level of visual spatial abilities get enhanced, whereas the high-level face perception, motion processing, and oculomotor behavior tend to be adversely affected suggesting that they are intrinsically linked to the binocularity (10–12). These differential effects may be due to a number of factors, such as plasticity through recruitment of resources to the remaining eye; the absence of binocular inhibitory interactions; and/or years of monocular practice (10). Aside from visual system effects, the monocular deprivation recruits cross-modal adjustments in the auditory system that support improved sound localization and integrates auditory and visual components of multisensory events optimally (13, 14). Structurally, people with one eye show an asymmetry in morphology of the anterior visual system, with an overall decrease in the lateral geniculate nucleus volume (15). In addition, early monocular enucleation not only increases the surface area and gyrification in visual, auditory, and multisensory cortices, but also has long-term effects on WM structure in the visual pathway and auditory tracts subcortically (16–19). Mechanisms that could support such morphological changes subsequent to long-term survival from early eye loss include Wallerian degeneration, neural recruitment of deafferented cells, corticothalamic feedback, and synaptic pruning (17). To date, however, alterations in functional networks at the whole-brain level remain largely unknown in early monocular deprivation.

Resting-state functional MRI (rs-fMRI) based on blood oxygenation level-dependent (BOLD) signals has been successfully applied to investigate the synchronous activity between brain regions and identify intrinsic large-scale networks (20). Earlier rs-fMRI studies divided human cortex into six macro-scale networks: the visual network (VN), somatomotor network (SMN), default mode network (DMN), attention network, control network, and salience network (21). Among different methods for rs-fMRI analysis, the amplitude of low-frequency fluctuation (ALFF) reflects the regional spontaneous brain activity at each voxel and the functional connectivity (FC) measures brain connectivity patterns between BOLD signals in different brain regions (22–25). The advances in the graph theory and network neuroscience afford an opportunity to study local and global topological properties of complex brain networks. Specifically, the brain is conceptualized as a graph, in which brain regions represent nodes and the relationships between the regions signify edges connecting the nodes within the graph (26, 27). A graph theory based-analysis can assess the importance of brain regions at the network level and evaluate its role in brain information transfer and integration. Thus, the above methods are complementary to each other.

It is noteworthy that the majority of previous studies have been done on individuals who have lost one eye during the postnatal visual development or later in life, which is inhomogeneous with respect to age of sensory deafferentation and visual experience. Congenital microphthalmia is a rare anomaly due to arrest of ocular growth and development in the early fetal life and affects 3–11% of blind children (28). Unilateral congenital microphthalmia offers a novel and unique human model for exploring the consequences of absence of binocularity and the extent to which the brain's functional networks rely on experience-dependent mechanisms since the brain is prenatally deprived of half the normal visual input (29). This study aimed to examine the changes of intrinsic neuronal activities and functional connectivity patterns, and further explore the graph-theoretical characteristics of functional brain networks in patients with unilateral congenital microphthalmia.

## Experimental Procedure

### Participants

The study adhered to the tenets of the Declaration of Helsinki and was approved by the Research Ethics Committee of Beijing Tongren Hospital, Capital Medical University. Informed written consent was obtained from all participants. Sixteen patients (eight women; age 20.81 ± 4.85 years) with unilateral isolated congenital microphthalmia (eight right and eight left) were recruited from the Beijing Tongren Eye Center. Exclusion criteria included age <16 or > 40 years, any ocular disorder other than congenital microphthalmia, any neurological disorder (documented clinically and instrumentally), and left-handedness. Sixteen healthy subjects (7 women; age 24.13 ± 3.50 years) with matched age, gender, and education status were recruited among students and workers from university and hospital as normal controls. All controls were right-handed and had normal neurological and ophthalmological examinations without history of neurological or ophthalmological disorders.

### Data Acquisition

MRI data were acquired using a 3.0-T MR scanner (Discovery MR750; General Electric, Milwaukee, WI, USA), with an 8-channel head coil. Initially, a 3-dimensional (3D) brain volume T1-weighted anatomical scan was conducted with the following parameters: repetition time (TR) = 8.16 ms, echo time (TE) = 3.18 ms, inversion time (TI) = 450 ms, flip angle = 12 degrees, matrix = 256 × 256, thickness = 1.0 mm, gap = 0 mm, 188 slices, and voxel size = 1.0 × 1.0 × 1.0 mm. A BOLD-fMRI scan was then performed using a gradient-echo single-shot echo planar imaging (EPI) with the following parameters: 36 axial slices, slice thickness = 3 mm, gap = 1 mm, TR = 2,000 ms, TE = 30 ms, flip angle = 90 degrees, field of view = 220 mm × 220 mm, matrix = 64 × 64, and 180 time points. During the BOLD-fMRI scan, all subjects were instructed to keep their eyes closed in a relaxed state, refrain from movement, and stay awake without concentrating on anything in particular.

### Pre-processing

Preprocessing of rs-fMRI data was performed with a public available toolbox for Data Processing and Analysis for Brain Imaging (DPABI, http://rfmri.org/DPABI) software package (30), running on a matrix laboratory platform named as MATLAB R2016a (https://www.mathworks.com/products/matlab.html, The MathWorks, Inc., Natick, MA, USA). The procedures included DICOM-to-NIFTI format conversion, removal of first 10 volumes, slice timing correction, head motion correction, nuisance covariates regression, standard space normalization with resolution of 3 × 3 × 3 mm and bounding box of [−90, −126, −72; 90, 90, 108], spatial smoothing with Gaussian kernel of 4 × 4 × 4 mm full-width at half maximum (FWHM). Any participant with a head motion more than 1.5 mm translation or 1.5°rotation on any axis was excluded. The nuisance covariates include head motion, respiratory, and cardiac effects (30). Head motion could introduce artifactual inter-individual difference in R-fMRI metrics (31, 32). The head motion effects from realigned data were regressed out by Friston 24-parameter model (33). The respiratory and cardiac effects were reduced through regressing out the signals from WM and cerebrospinal fluid (CSF).

### Flip Images

Considering the potential confounding effects of affected sides, we swapped the hemisphere of the left affected patients making the affected sides of all subjects kept in right. The flip was implemented via flip_lr.m of a MATLAB toolbox called “Tools for NIfTI and ANALYZE image.” Therefore, the right side is ipsilateral side of affected eye, whereas the left is contralateral side.

### ALFF Analysis

Amplitude of low frequency fluctuation measures the total power of the BOLD signal within a certain low-frequency range and has been widely applied to study the regional brain activity and functional segregation (34). The brain areas with a high ALFF may correspond to an increased spontaneous neuronal activity. The fast Fourier transform was used to compute ALFF as the average of the power spectrum's square root in the 0.01–0.08 Hz frequency bandwidth. The ALFF value of each voxel was further standardized by z-score to obtain the zALFF value. The two-sample *t*-test within gray matter mask was conducted to compare the zALFF values between two groups with age and gender as covariates. The Alphasim (*p* < 0.005 and number of voxels > 37 of 26 Neighborhood) embed in DPABI was used for correction.

### Region of Interest-Based FC Analysis

Functional connectivity refers to the temporal correlation in spontaneous BOLD fluctuations between brain regions and may reflect the inter-regional correlations in neuronal variability. The altered brain regions (clusters with significant ALFF differences between microphthalmic subjects and controls) were selected as seed regions of interest (ROIs) for FC analysis. For each ROI of each subject, correlation coefficients between the mean time series of the seed ROI and the other ROIs were computed. The correlations were then converted into z scores by Fisher's *r*-to-*z* transformation to normalize the FC values by DPARSF in DPABI. The two-sample *t*-test was applied to compare the correlations of ROIs between patients with congenital microphthalmia and normal controls. To visualize the FC values on each ROI of each group, the mean FC of the ROI and the other ROIs were computed.

### Graph Theory-Based Network Analysis

GRETNA (www.nitrc.org/projects/gretna/), a Matlab-based, open-source graph theoretical network analysis toolbox for imaging connectomics, was employed to construct the functional brain network (35). Various topological properties of a network or graph were calculated from global metrics (small world, network efficiency, rich club, assortativity, synchronization, and hierarchy) and nodal metrics (clustering coefficient, shortest path length, nodal efficiency, local efficiency, degree centrality, and betweenness centrality). The pipeline options were as follows: absolute FC values were applied to construct the binarized networks; sparsity was selected as the threshold metrics with the thresholds ranging from 0.05 to 0.5 (interval = 0.05); random network generation number was set to 100; for the community index, the modularity algorithm was set to modify greedy optimization. The network sparsity was chosen as a thresholding method.

## Results

### Intergroup Differences of ALFF

**Figure 1:**
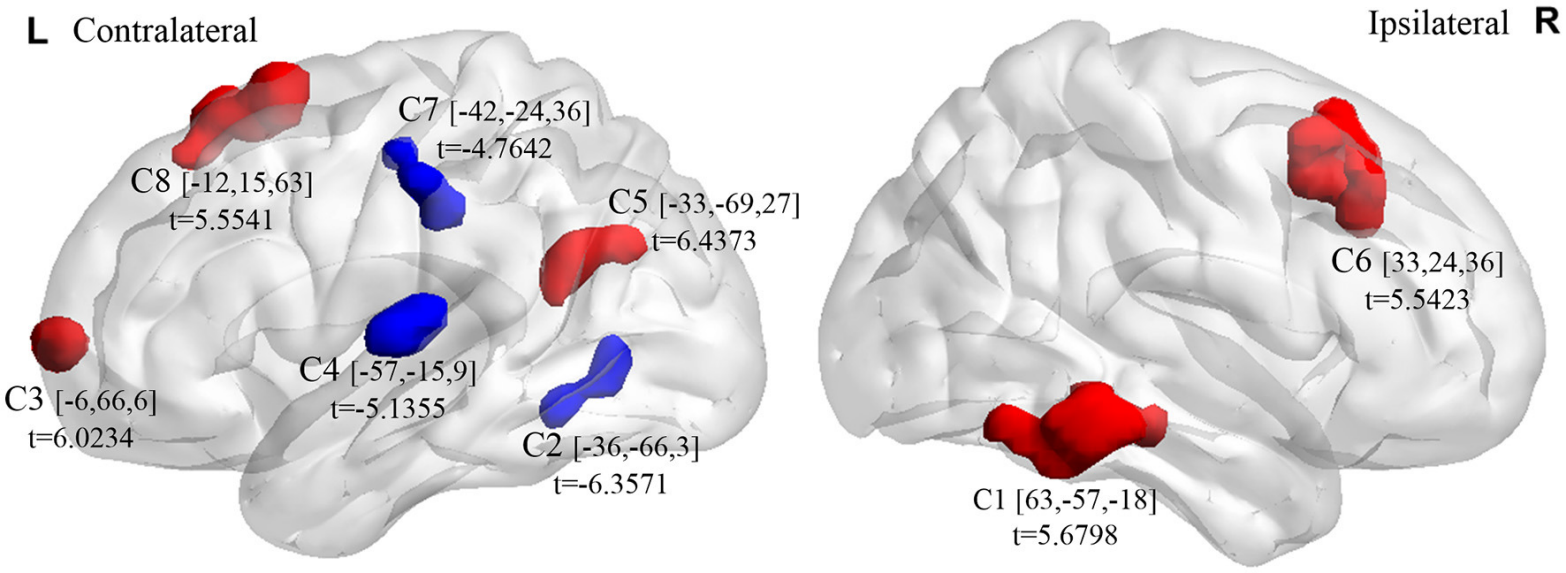
Brain clusters (C1–C8) exhibiting intergroup differences in ALFF between congenital microphthalmia (CM) and normal controls (NC). Blue color indicates decreased spontaneous neuronal activity, whereas red color indicates increased spontaneous neuronal activity. The peak *t*-values and the corresponding MNI coordinates were presented near the clusters. ALFF, amplitude of low-frequency fluctuation. MNI, Montreal Neurological Institute.

Compared with the control group, the microphthalmia group had significantly altered ALFF in the 8 brain clusters (C1–C8) (Alphasim correction with *p* voxel < 0.005, number of voxels > 37) (Figure 1, Table 1). The patients with congenital microphthalmia showed decreased ALFF in the C2 encompassing left inferior occipital gyrus and inferior temporal gyrus (BA37), C4 located in the left superior temporal gyrus (BA22/48), and C7 encompassing left inferior parietal lobule and post-central gyrus (BA3). On the other hand, increased ALFF was found in the C1 encompassing right middle temporal gyrus (BA21) and inferior temporal gyrus (BA20), C3 located in the left medial superior frontal gyrus (BA10), C5 dominated by left BA39 containing the angular gyrus in the superior temporal gyrus, C6 covering the right middle and superior frontal gyri (BA7/8/46), and C8 located in the left superior frontal gyrus, such as supplementary motor area (BA6/8).

**Table 1:** Brain regions with intergroup differences in amplitude of low-frequency fluctuation (ALFF) between congenital microphthalmia (CM) and normal controls (NC). Clusters 1–8 correspond to the 8 clusters in Figure 1.

**Brain regions**	**# voxels**	**Coordinates** **in MNI**	**Peak** ***t*-values**
**CM > NC**			
Cluster 1 (C1)	75	[63, −57, −18]	5.6798
BA20_R/BA21_R	36/22		
Temporal_Mid_R (AAL)	34		
Temporal_Inf_R (AAL)	31		
Cluster 3 (C3)	50	[−6, 66, 6]	6.0234
BA10_L	41		
Frontal_Sup_Medial_L (AAL)	38		
Cluster 5 (C5)	55	[−33, −69, 27]	6.4373
BA39_L	39		
Angular_L (AAL)	18		
Superior temporal gyrus	17		
Cluster 6 (C6)	96	[33, 24, 36]	5.5423
BA8_R/BA7_R/BA46_R	34/24/14		
Frontal_Mid_R (AAL)	51		
Frontal_Sup_R (AAL)	43		
Middle frontal gyrus	54		
Cluster 8 (C8)	80	[−12, 15, 63]	5.5541
BA6_L/BA8_L	34/26		
Supp_Motor_Area_L (AAL)	39		
Superior frontal gyrus	68		
**CM < NC**			
Cluster 2 (C2)	53	[−36, −66, 3]	−6.3571
BA37_L	48		
Temporal_Inf_L (AAL)	10		
Occipital_Inf_L (AAL)	10		
Cluster 4 (C4)	38	[−57, −15, 9]	−5.1355
BA22_L/BA48_L	15/17		
Temporal_Sup_L (AAL)	37		
Cluster 7 (C7)	37	[−42, −24, 36]	−4.7642
BA3_L	16		
Parietal_Inf_L (AAL)	15		
Postcentral_L (AAL)	12		

*The location of each cluster was defined via the automated anatomical labeling (AAL) atlas and Brodmann area (BA). MNI, Montreal Neurological Institute*.

### Intergroup Differences of ROI-Based FC

**Figure 2:**
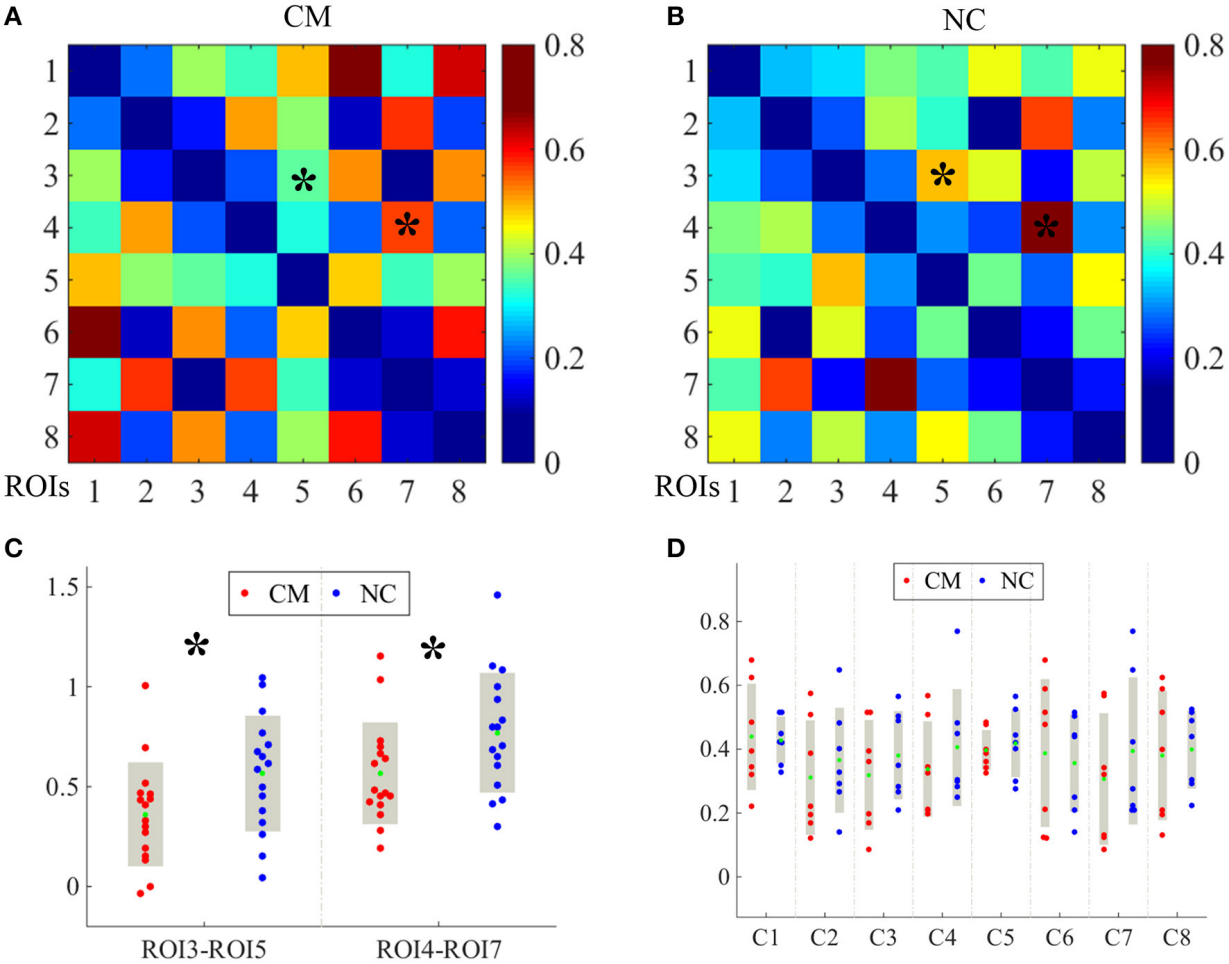
The averaged region of interest (ROI)-based functional connectivity (FC) values after Fisher's r-to-z transformation of CM and NC groups were shown as in image **(A,B)**, respectively. Each cluster was taken as one ROI for FC computation. The FC values between C3 and C5, and FC values between C4 and C7 [indicating by * in image **(A,B)**] were significantly altered between CM and NC. The significantly reduced FC values were presented in image **(C)**. The FC values between all 8 clusters of CM (red dots) and NC (blue dots) **(D)**. The green dots indicate mean FC values. *p < 0.005.

The averaged ROI-based FC values after Fisher's *r*-to-*z* transformation of CM and NC are shown as in Figure 2. Compared with the control group, the microphthalmia group had significantly declined FC between the left medial superior frontal gyrus (C3) and angular gyrus (C5), as well as FC between left superior temporal gyrus (C4) and inferior parietal lobule, post-central gyrus (C7) (*p* < 0.005). The changes indicate weakening of information transmission between these brain regions.

### Intergroup Differences of Network Properties

**Figure 3:**
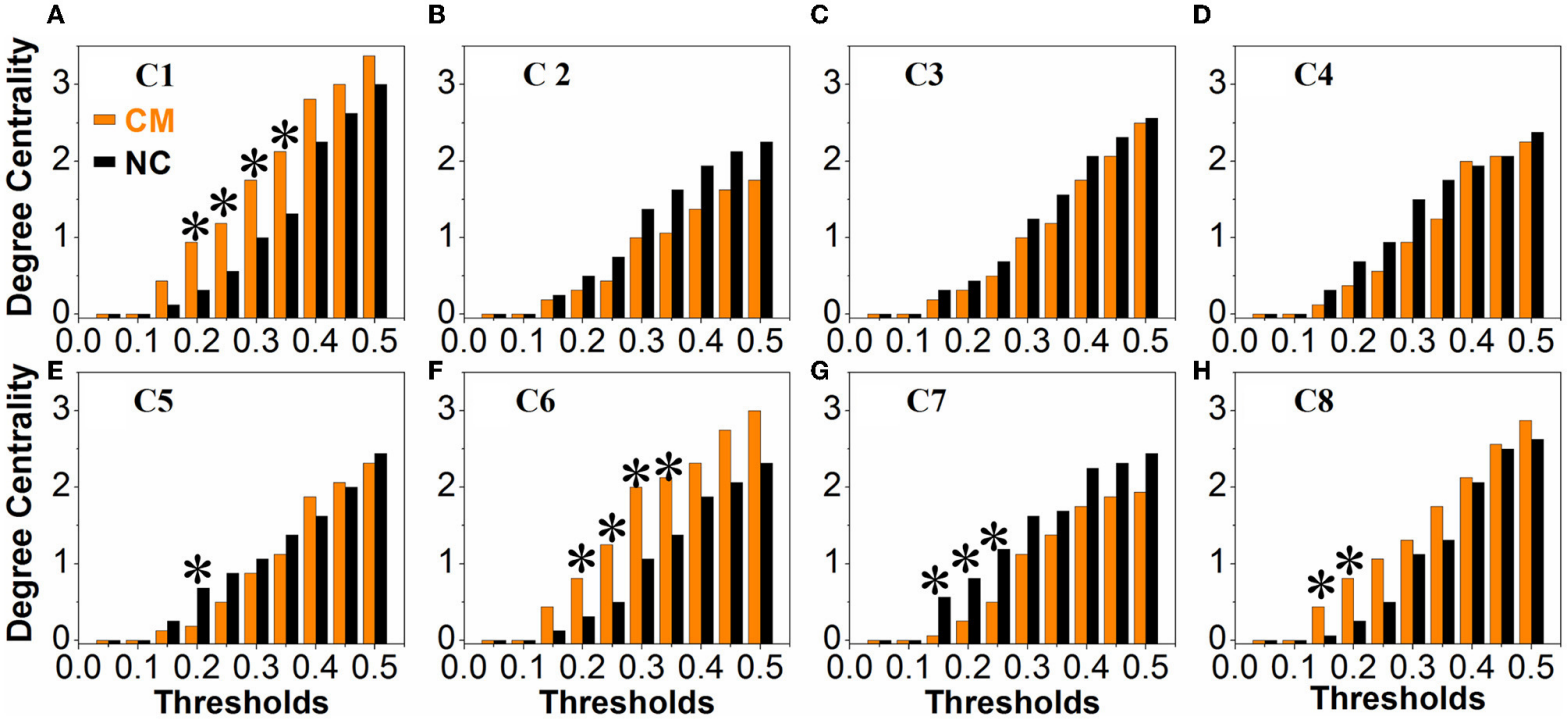
The CM group showed significantly increased degree centrality values on C1 (at thresholds of 0.2, 0.25, 0.3, 0.35), C6 (at thresholds of 0.2, 0.25, 0.3, 0.35), and C8 (at thresholds of 0.15 and 0.2), and decreased degree centrality values on C5 (at thresholds of 0.2) and C7 (at thresholds of 0.15, 0.2, and 0.25). *p < 0.05. **(A–H)** All the vertical coordinates of subfigures are degree centrality, and all the horizontal axis were thresholds. The symbol * at each subfigure indicates that the degree centrality of the cluster was significantly changed at the corresponding threshold.

**Figure 4:**
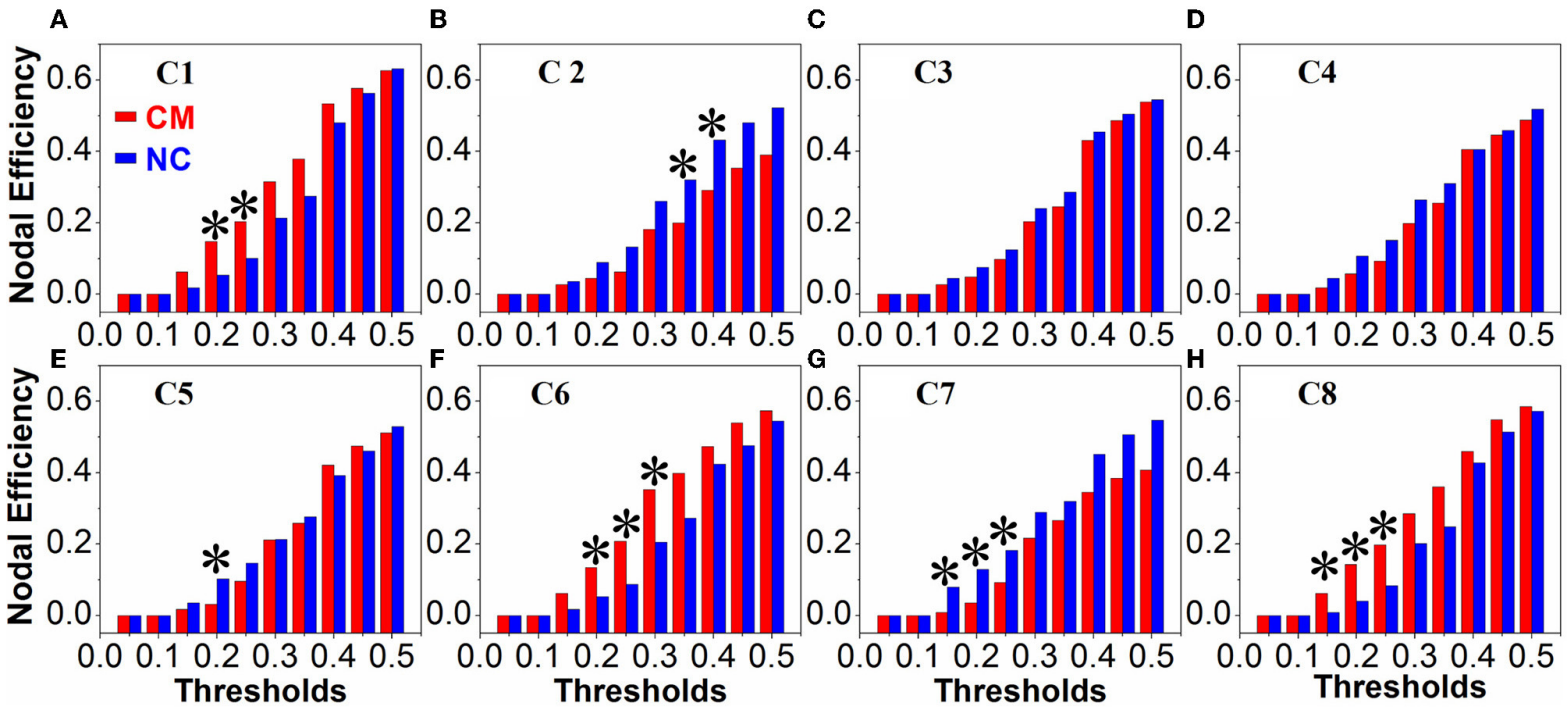
Nodal efficiency values of CM significantly increased on C1 (at thresholds of 0.2 and 0.25), C6 (at thresholds of 0.2, 0.25, and 0.3), and C8 (at thresholds of 0.15, 0.2, and 0.25), and significantly decreased on C2 (at thresholds of 0.35 and 0.4), C5 (at threshold of 0.2) and C7 (at thresholds of 0.15, 0.2, and 0.25). *p < 0.05. **(A–H)** All the vertical coordinates of subfigures are nodal efficiency, and all the horizontal axis were thresholds. The symbol * at each subfigure indicates that the nodal efficiency of the cluster was significantly changed at the corresponding threshold.

There were no obvious differences between the microphthalmia group and control group in terms of global network metrics. As for the regional network metrics, significant differences were found in degree centrality (Figure 3) and nodal efficiency (Figure 4; *p* < 0.05). Degree centrality calculates the number of direct connections a given node has, reflecting its information communication ability in the functional network. Patients with microphthalmia displayed higher degree centrality values on C1, C6, and C8, whereas lower values on C5 and C7. Nodal efficiency measures the average shortest path length between a given node and all of the other nodes in the network, which stands for the ability of the node in communication transfer within a network. Nodal efficiency values of patients with microphthalmia increased on C1, C6, and C8, while reduced on C2, C5, and C7.

## Discussion

The present study investigated the intrinsic neuronal activity and network topological properties across the whole brain in patients with congenital monocular blindness. Unilateral congenital microphthalmia represents an exceptional opportunity to explore experience-dependent reorganization when deprived of half natural inputs prior to birth. The unique visual experience plays an important role in shaping neural circuits, function, and ultimately behavior. The findings may improve our understanding of how early monocular deprivation influences the brain development.

Patients with microphthalmia exhibited attenuated spontaneous brain activities in the left inferior occipital and temporal gyri, superior temporal gyrus, inferior parietal lobule and post-central gyrus when compared with healthy subjects. The inferior occipital and temporal gyri lie within the ventral stream of visual processing known to be associated with object recognition (“what” pathway) (36, 37). A strong contralateral retinotopic bias was confirmed present throughout the occipitotemporal network (36). Meanwhile, the inferior occipital gyrus and the fusiform gyrus (BA37) of the inferior temporal gyrus are identified as face-specific regions (38). Face perception is a unique visual ability which is considered anatomically and functionally at higher level and critical for normal social functioning. Kelly et al. demonstrated that early monocular deprivation from enucleation selectively disrupts the neural development of face perception and thus impair face processing (11, 12). In addition, left BA37 is involved in visual-language associations, such as semantic categorization, word retrieval, and word generation (39). Superior temporal gyrus is the site of auditory association cortex and the left part is considered as a shared substrate for auditory short-term memory and speech production (40). The inferior parietal lobule is a multimodal association area which situated at the junction of the visual, auditory, and somatosensory cortices. It contributes to aspects of receptive language, such as phonology, reading, and spelling, particularly in the language-dominant hemisphere. The post-central gyrus corresponds to the primary somatosensory cortex and it perceives various somatic sensations from the body, such as touch, pressure, temperature, and pain. Previous studies have uncovered robust correlation between the visual cortex and post-central gyrus in sighted people, which was involved in the combining process of spatial visual and somatosensory information (41).

On the contrary, enhanced intrinsic brain activities were observed in a set of brain regions of patients with congenital microphthalmia. The middle and inferior temporal gyri subserve language and semantic memory processing, visual perception, and multimodal sensory integration. The angular gyrus (BA39) situates at the junction among the occipital, temporal, and parietal lobes and resembles a cross-modal integrative hub linking different subsystems (42). In addition to being associated with semantic processing, the left angular gyrus has been identified as part of the default network (43). The medial superior frontal cortex is implicated in motor and cognitive control, among which the eye movement is thought to be a critical vision-related function (44, 45). The supplementary motor area (BA6) occupying the posterior one-third of the superior frontal gyrus is responsible for planning of complex movements of contralateral extremities and also suggested to have superordinate control functions during speech communication and language reception (46, 47). The middle frontal gyrus is regarded as a key integration cortical hub for both dorsal and ventral streams of language (48). Adjacently, the frontal eye field (BA8) plays an important role in the control of visual attention and saccadic eye movements.

Our results showed increased average capability of information communication (degree centrality) and efficiency of information transfer (nodal efficiency) in regions, such as middle and inferior temporal gyri and middle and superior frontal gyri of the microphthalmic patients. However, these capabilities declined in the regions, such as inferior occipital and temporal gyri, inferior parietal lobule, post-central gyrus, and angular gyrus. From the above, brain regions with more importance in the brain functional network of patients with congenital monocular blindness predominantly belong to the speech and language neural network.

Potential limitations of the current study should be noted. First, as a pilot study, neuropsychological tests and visual tasks have not been included. Despite the fact that changes detected in the regions are associated with cognitive functions, it is difficult to confirm whether these altered ALFF values are correlated with cognitive changes. A visual task is vital for the future work to capture the characteristics of local brain activities during visual processing of congenital microphthalmia. Second, the sample size was modest and our results require replication with larger sample sizes.

In conclusion, individuals with unilateral congenital microphthalmia presented remarkable alterations of regional brain functions mainly in the vision and language related networks. Our data shed light on functional reorganizations of the brain network induced by congenital monocular blindness and lend further support to the involvement of cross-modal plasticity.

## Data Availability Statement

The original contributions presented in the study are included in the article/supplementary material, further inquiries can be directed to the corresponding author/s.

## Ethics Statement

The studies involving human participants were reviewed and approved by Beijing Tongren Hospital, Capital Medical University. Written informed consent to participate in this study was provided by the participants' legal guardian/next of kin.

## Author Contributions

JDi and XQ contribute to designing, interpreting, and drafting the work. JC, JDo, and JG contribute to the acquisition of the work. JX and DL contribute to designing and revising the work. All authors contributed to the article and approved the submitted version.

## Funding

This study was supported by grants from the Special Fund of the Pediatric Medical Coordinated Development Center of Beijing Hospitals Authority (XTCX201824), the priming scientific research foundation for the junior research of Beijing Tongren Hospital, Capital Medical University (2017-YJJ-GGL-004 and 2018-YJJ-ZZL-011), Beijing Municipal Administration of Hospitals' Ascent Plan (DFL20190203), and the National Natural Science Foundation of China (82071005, 82071906, 81701666, 81871340, and 81901719).

## Conflict of Interest

The authors declare that the research was conducted in the absence of any commercial or financial relationships that could be construed as a potential conflict of interest.

## Publisher's Note

All claims expressed in this article are solely those of the authors and do not necessarily represent those of their affiliated organizations, or those of the publisher, the editors and the reviewers. Any product that may be evaluated in this article, or claim that may be made by its manufacturer, is not guaranteed or endorsed by the publisher.
